# Interaction Rituals at Content Trade Fairs: A Microfoundation of Cultural Markets

**DOI:** 10.1177/08912416221113370

**Published:** 2022-08-03

**Authors:** Andreas Gebesmair, Christoph Musik

**Affiliations:** 1 Media Business Research Group, St. Pölten University of Applied Sciences, St. Pölten, Austria; 2 Acker Österreich gemeinnützige GmbH, Brunnenthal, Austria

**Keywords:** Randall Collins, interaction rituals, cultural industries, trade fairs, economic sociology, emotions

## Abstract

In this article, we show how ritualized periodic encounters of business partners help to reproduce business relations and a shared understanding of doing business based on ethnographic fieldwork at six international trade fairs in three different cultural industries. We draw on Randall Collins’ theory of interaction rituals (IRs), which highlights the relevance of emotional contacts in social life. Although Collins’ theory and his conceptional instruments help to shed light on a neglected aspect in the sociology of markets, our results go beyond his ethological interpretation of interactions. First, we conclude that Collins underestimates the direct impact of the uneven distribution of economic resources on IRs. Second, we observed not only emotional entrainment in IRs but also the strategic production of emotions.

## Introduction

Corporate buyers usually do not buy goods and services on anonymous markets. They buy from sellers they have known personally, often for years, and to whom they maintain stable relations. These partners inform each other about their goods, business practices and new technologies, they help each other in assessing products, and they share fundamental beliefs regarding what their business is about and how it should be pursued. These markets often rest on long-standing, trustful ties and taken-for-granted rules, which help to reduce uncertainty and transaction costs. They are, as economic sociologists put it, socially and culturally embedded (DiMaggio 1994; Granovetter 1985).

This holds true, in particular, for the media und cultural industries, which are said to be a “people’s business” (Hesmondhalgh and Baker 2011; Roussel 2015; Sydow and Windeler 2004), where continuous contact and personal networks play an important role. Sellers and buyers on local as well as global media markets do not only maintain close relationships, they also share a common culture of doing and understanding their business. For example, Kuipers (2011) discovered in her study on buyers in the global TV industries a highly networked group of transnational intermediaries with a shared professional ethos and similar practices (see also Havens 2011; Moeran and Pedersen 2011).

But where do these strong ties and shared cultures among global cultural intermediaries come from and how are they reproduced? Following Randall Collins’ radical microfoundation of macrosociology, we assume that ritualized periodic encounters of business partners help to establish, confirm and transform those networks and belief systems, which are constitutive for markets. It is not only the reciprocity of expectations and mutual trust that keeps markets together, there is also an emotional underpinning of economic action (Bandelj 2009, 2015; Berezin 2009; Pixley et al. 2014), which is of great importance. As Randall Collins has shown, people are bound to each other and to cultural symbols by emotional energy (EE) (Collins 2004). Hence, relations between business partners have to be ritualized in order to provide a solid basis for market transactions.

In this article, we discuss the role of interaction rituals (IRs) in cultural markets. Based on ethnographic fieldwork at six international trade fairs in three different cultural industries, we show how business relations and a shared understanding of doing business are reproduced through IRs. Trade fairs provide a perfect frame for highly ritualized and emotionalized encounters, since they bring together a large number of business partners during a short and intense event, which stands out against the daily routines of business life. But they are also an arena where economic actors with different degrees of market power strategically assert their interests. Although Collins considers IRs as crucial for social stratification, we think that his exclusive focus on EE and emotional entrainment ignores other important aspects of interactions that are relevant in business environments. Therefore, we propose a conceptual extension of Collins’ approach that reconciles his preoccupation with emotions with a more traditional view of economic markets, in which power relations and strategic action play an important role.

The article is split into four main sections. In the first part, we briefly discuss Randall Collins’ contribution to the sociology of commercial markets against the background of recent work on rituals in economic life. We touch on central elements in his theory and develop a framework for analyzing IRs on content trade fairs. In the second part, we introduce our methodological approach and point to some characteristics of ethnographies at trade fairs. After presenting detailed results from fieldwork in the third part, we finally sum up our main findings which go beyond Collins’ conception and may help to extend his theory. We conclude with a short summary and some recommendations for future work.

## Toward a Microfoundation of Cultural Markets

### Rituals in Economic Life

Rituals in economies became an important subject of research in recent years. Especially scholars of management highlighted the relevance of large business gatherings such as festivals, conferences, and trade fairs for maintaining and transforming the institutional structure of an industry. Since these events assemble large numbers of field participants for intense interaction, and since they enjoy high legitimacy in the whole field, they are a forum for collective sensemaking, that is, “setting standards, defining practices, and codifying key vocabularies” in an industry (Lampel and Meyer 2008, 1029). Research has shown that they helped to establish new product categories on music and book markets (Anand and Jones 2008; Anand and Watson 2004), to introduce new narratives for regulating persistent organic pollutants (Hardy and Maguire 2010) or to set a political agenda in the music business (Dobusch and Schüßler 2014), to cite just a few of examples.

The ritual and ceremonial character of these so-called field-configuring events are crucial. Picking up insights from different schools of ritual research, Anand and Watson (2004) analyzed business rituals as a medium of conflict upon the visibility and legitimacy of new market categories. Since award ceremonies, such as the Grammy awards of the music industry, simultaneously interlock disparate parts of the industry and direct their attention to a common “sacred object,” that is, the most valued music productions of a year, they are repeatedly used by marginalized groups to introduce, and advocate, neglected genres such as Rap or Latin music in the 1990s. Thus, tournament rituals, as Anand and Watson call this form of business events, became an important instrument for transforming the music industry.

While research on business events by management scholars mainly focuses on the macro-level outcomes of rituals, economic sociologists inspired by Vivienne Zelizer’s seminal work on economic lives concentrate on the micro levels of business interactions. Ethnographic research conducted by Zelizer and her colleagues has shown that economic transactions often occur within circuits, which have to be nurtured by interaction partners through relational work (Bandelj 2015; García 2014; Zelizer 2010, 303–53, 2012). Circuits consist of distinctive social ties for a defined set of transactions and certain transaction media, whose meaning and moral valuation are constantly contested and renegotiated. Although Zelizer developed her analytical instruments in studies on morally precarious and exposed areas of the economy, for instance intimate transactions or life insurances, she insists that most commercial markets require relational work. It makes market transactions meaningful and draws boundaries between legitimate and non-legitimate transactions. Again, rituals play an important role in defining these boundaries. In his study on craft markets in Thailand, Wherry (2012) has shown how nonnative commercial sellers and buyers of so-called Thai spirit houses (i.e., indigenous furniture which has a spiritual function but is also bought as a lifestyle accessory) must run through a series of negotiations and dramaturgical performances which transform a sacred object into a commercial good that can be sold for profane purposes. “The negotiations are not about the final price but rather about how the real spirit houses would need to be decommissioned properly in a ritual led by the monks before they could be sold (. . .) The buyer can purchase a sacred object only after honoring the object’s sacred, ritual character” (Wherry 2012, 211). Similar forms of a performative transformation of a highly valued object to a priced commodity can be found in the art market or in the rebranding of urban ethnic economies (Wherry 2012).

The most elaborated perspective on rituals in economic life (and society in general) is doubtlessly Randall Collins theory of Interaction Ritual Chains. In contrast to the aforementioned approaches, Collins applies his theory to all forms of interactions—profane as well as the sacred, business as well as nonbusiness—and highlights the emotional underpinning of these rituals. Drawing on Durkheim’s and Goffman’s fundamental insights into the ritual basis of traditional as well as modern societies, Randall Collins developed a radical microsociology of social structure over several decades of research on a great diversity of different subjects (summed up in Collins 1981, 1988, 2004; Collins and Sanderson 2009). In contrast to other microsociological approaches, he built his theory not on the human capacity of arriving at a shared interpretation of situations but on the rhythmic coordination and synchronization of conversational partners, which occur in all interactions not only between humans but also between animals. In his view, it is not mutual trust in meaningful behavior that establishes the order in interactions but emotional entrainment and the affective binding of interacting partners to each other and to the situation. Entrainment means that interaction partners arouse emotions in each other and thereby provide a kind of subliminal basis for coordination. For Collins everyday interactions in contemporary societies fulfill the same function as religious ceremonies or other rituals in traditional societies: they evoke strong emotions, “collective effervescence” in Durkheim’s words, and a feeling of solidarity. Simultaneously, they bind us to symbols (“sacred objects”), in which this solidarity is expressed (Collins 2004). This seems to be one of Collins most crucial aspects of his theory: the symbolic order of a society must be emotionally consolidated to make it durable. In IRs, symbols get effectively loaded and, hence, become a medium, and storage of solidarity and social bonding. Whenever people get together in co-presence with a shared mood and a mutual focus of attention, they transfer EE and bind themselves to symbols and to each other.

From this perspective, IRs in economic life and material markets play the same role as IRs in other parts of the society (Collins 1993, 2004, 81–7, 141–82): on one hand, they reinforce the mutual trust of business partners, which is crucial for avoiding excessive transaction costs, and, on the other hand, they provide business people with those taken-for-granted beliefs and routines, which are a prerequisite for competently navigating through a complex and uncertain business environment. At trade fairs and other business events, businesspeople talk a certain jargon, inform each other about new trends, learn about business practices, and discuss recent changes in the industry.^1^ Since this complex of business lore, buzz, and material practices evolves from rituals where people entrain each other emotionally, it becomes broadly institutionalized. In contrast to the approaches discussed earlier in this article, Collins conceives the institutional order of markets not so much as the outcome of deliberate negotiations of meaning, but as the consequences of a ritual situation where business partner invest different amounts of EE and affectively bind others and themselves to certain symbols.

In the next section, we outline how research on business events in cultural markets can benefit from his theory. We carve out two aspects of Collins approach which seem especially useful for analyzing IRs at content trade fairs in the cultural industries: the sociable and formal character of business events and their contribution to stratification.

### A Framework for Analyzing IRs at Business Events

Business events such as trade fairs have all ingredients of an IR as outlined by Collins (Collins 2004, 48; Collins and Sanderson 2009, 58): There is a group assembled in bodily copresence,^2^ which has a mutual focus of attention and a shared mood. Furthermore, there is a clear barrier to outsiders, which is established not only by considerable access fees but also by an abundance of knowledge and cultural resources that are required to competently perform at these events. However, IRs at business events differ substantially from context to context. We touch on two dimensions that seem crucial in classifying IRs: the degrees of sociability and the degrees of formalization (Collins 2004, 49–53, 268–78).

*Sociability and formalization*. In his early work on conflict sociology, Collins distinguishes several types of conversation (Collins and Sanderson 2009[1975], 72–86). The list comprises practical talk in work contexts and intellectual discussions as well as personal talk, gossip, and entertainment talk. These different types of interaction do not only fulfill different (manifest) functions in daily life, they also differ in their emotional tones. While negotiations of contracts usually take place in a formal atmosphere, small talk at parties is mostly embedded in a relaxed mood and pleasant sentiments. But the main difference lies in the varying purposes of these interactions: While practical talks or economic transactions focus on goals beyond the interaction, small talk at parties is an end in itself. As Georg Simmel showed a century ago (Simmel and Hughes 1949), *sociability* is the purest form of association between humans, and the more these associations are burdened by individual interests and external purposes, the more they lose their character of a free conversation between equals.^3^ Spontaneous sociability between strangers that is widespread in public life (see, e.g., Anderson 2011; Horgan et al. 2020) does not occur frequently at trade fairs, where economic interests and business goals permeate almost all interactions. Nevertheless, there are some occasions when business partners forget about doing business and enjoy the pure pleasure of a sociable conversation. Thus, economic transactions and parties should be regarded as extremes on a continuum on which external purposes and sociability amalgamate in different ratios of mixtures.

IRs at business events differ also in the degree of *formalization*. They are usually hardly scripted. In contrast to religious ceremonies, there is no recitation of verbal formulas, there are no stereotyped gestures, or traditional costumes. Nevertheless, they are highly routinized and show some degree of standardization. Anand and Jones (2008), for example, reconstruct from media coverage of the annual Booker Prize ceremony the ritual scripts that steer this business event. As we will see, IRs at trade fairs are also formalized to some degree. They take place within a restrictive temporal and spatial structure. Of course, there is space for spontaneous encounters and improvised action (natural rituals in Collins’ terms). Nevertheless, especially buyers and sellers have a tight schedule and navigate through an almost perfect spatial order. Furthermore, highly standardized ceremonial elements have developed at some events over the years.

**Figure 1.**
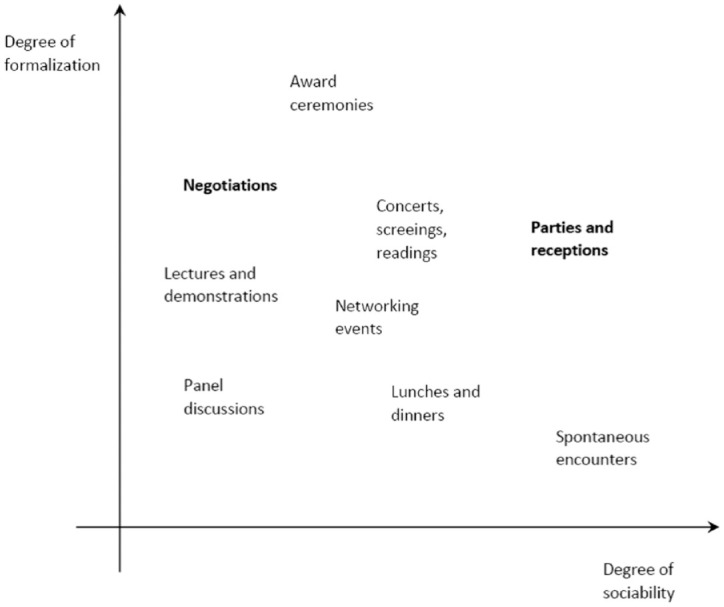
IRs on Trade Fairs.

The degrees of sociability and the degrees of formalization do not vary independently. Collins (2004, 272) assumes that sociability is highest in situations that are neither too formal nor too informal (see also Simmel and Hughes1949). People usually feel most comfortable in gatherings that are to some degree focused, scripted, and scheduled, but not too much. Nevertheless, at content trade fairs, we find IRs that blend formalization and sociability in many different ways. Figure 1 shows an overview of different forms of encounters, which range from those that are highly formalized, as for instance negotiations, to the ones that are sociable spontaneous encounters, for example, at the trade fair floor. We will elaborate on these IRs in the results section.

#### Stratification

We want to touch on another aspect of IRs that was studied by Collins during his whole academic career and that is also relevant for cultural markets: *stratification*. Beginning with his early work on conflict sociology (Collins and Sanderson 2009[1975]), which builds mainly on Max Weber’s theory of status groups, Collins shows how inequalities in society are produced, reproduced, and converted through status and power rituals in chains of interactions (see also Collins 2004). In *status rituals*, members of a status group assure themselves of their distinctiveness through specific manners and mutual deference. Central to all status rituals is deciding who is a member and who is not. In contrast, *power rituals* focus on the process of giving and taking orders between unequal interaction partners, which reproduces the degree of subordination. In real-life encounters, status and power effects of IRs often occur simultaneously, since we usually observe some hierarchical stratification within status groups, and forms of solidarity and identity even across groups in distant strata of society (e.g., in nation states).

We can find status and power rituals also at business events such as trade fairs where business partners of different status and power meet with each other. Of course, most rituals have status effects. The numerous conversations at the booths, the business meetings in lounges, noble restaurants and hotel suites, the parties and receptions, and the public presentations doubtlessly serve to foster solidarity and identity within the industry as a whole, or in fractions of it, and build up social and symbolic barriers against all others. Simultaneously, there are also power rituals that confirm or renegotiate structures of subordination. This holds true not only for bargaining a deal, which is per definition a ritual that results in a vertical stratified market position but also for interactions between incumbents and newcomers or executives and their assistants and other subordinates.

In Collins’ view, the most important element in maintaining and renegotiating social order is EE. A positive emotional disposition, a feeling of self-confidence, and the power to entrain others are important resources in status and power rituals. Together with cultural resources (i.e., a familiarity with relevant routines, knowledge, and symbols), EE mainly defines the outcome of an IR. The more people are able to impress others in personal encounters, the more they will succeed in future interactions. Thus, both cultural resources and EE are acquired, accumulated, and redistributed in chains of interaction and, thereby, affect social stratification. Although we see some problems in Collins’ conception of interaction resources (see discussion below), we address the question of how EE and cultural resources pervade status and power rituals at trade fairs in more detail in the results section.

## Methods

In a large comparative project on the global trade of cultural goods, we visited six different trade fairs in the book, the music and the TV industries between 2015 and 2017. The main goal of the project was to detect a common “trading culture,” that is, a cross-sectional institutional basis in the field of cultural production and distribution (see Gebesmair et al. 2022). When conducting fieldwork, we realized that the numerous highly emotional encounters and gatherings during these events play an important role in reproducing the shared culture. Thus, we shifted our focus from the social meaning of economic transactions to their ritual character. Interactions take place especially between buyers and sellers of content (more precisely of licenses and rights), who are the main (but not necessarily the most visible) actors at trade fairs. Buyers and sellers travel with their entourage of assistants, specialists, and artists, who together with industry representatives, suppliers of services, and the general public (which is permitted at some events) crowd on the trade fair floor and participate in IRs in one way or the other.

For each of the three industries, we selected a prominent, large fair that is representative of the whole industry and another one that represents only a specific sector of that industry. In the field of TV production, we visited the Marché International des Programmes de Communication (MIPCOM) in Cannes (France) and the European Film Market (EFM) in Berlin (Germany); for the music industry the Marché international de l’édition musicale (Midem) again in Cannes (France) and the Eurosonic Noorderslag in Groningen (The Netherlands); the book publishing industry was represented by the Frankfurter Buchmesse (Germany) and the Bologna Children’s Book Fair (Italy). Although these trade fairs differ substantially in size, target group, and character, they all provide a framework for a myriad of IRs that resemble each other across these industries and across the fairs. We will give an overview of different types of IRs on trade fairs in the next section.

Ethnographic research at business events poses some challenges. Although short-term ethnographies could be of a considerable intensity (Pink and Morgan 2013), which seems especially relevant when analyzing IRs, it is almost impossible to establish those trustful relationships that are usually required to access a field and fully participate in everyday economic practices (see, e.g., Abolafia 1998). During the four to six days of a trade fair, practitioners are extremely busy: They rush from one meeting or event to the other, they often hide in arcane areas for confidential negotiations, and they do not always feel comfortable when being addressed or accompanied by researchers. To mitigate this problem, we conducted 18 semi-structured interviews in advance, predominantly with buyers, but also with salespeople, consultants, and association executives from Austrian companies in all three cultural fields. These interviews did not only offer important insights into everyday practices at trade fairs but they also helped us to establish personal, even amicable ties to practitioners, which allowed a very intense and “emic” experience of the events. They provided us with inside knowledge, gave us hints on where to go, introduced us to other practitioners, opened doors to by-invitation-only events, and even let us take part in confidential negotiations—at least twice. Therefore, we had the opportunity to “shadow” the main actors in the field even across several sites and were led through these sites to those places that are relevant from the “natives’ perspective” (Delgado and Cruz 2014).

The fieldwork was conducted by a team of three sociologists, who were trained in ethnographic research. One researcher participated in all six trade fairs, and the other two visited three each. Our field notes included not only extensive descriptions of interactions but also methodological and theoretical reflections, and especially a detailed recording of our own feelings and emotions during participatory observation. In accordance with recent research on the role of extra-deliberate processes in day-to-day interactions, we believe that researchers’ emotions during fieldwork are reliable sources for the understanding of IRs and important building blocks in analytic ethnography (Summers-Effler, Ness, and Hausmann 2015). Although we do not always explicitly refer to these emotions in the following analysis, they played an important role in interpreting our ethnographic material.

## Results

### The Role of Content Trade Fairs in Cultural Markets

Trade fairs provide a framework for numerous status and power rituals in the cultural industries that differ in their degrees of formalization and sociability. We thoroughly describe and analyze these rituals in the next section. Before we go into details of IRs, we elaborate on the form and the role of content trade fairs in cultural markets in general.

As outlined in the theoretical introduction, IRs, in order to effectively work, require some degree of *formalization*. This holds true for trade fairs in general, which are simultaneously extraordinary and routine. They usually take place in huge, glamorous venues with a multitude of amenities and culinary pleasures on and off the site, and they stand out from everyday business at home during the rest of the year. They are, as Moeran and Pedersen (2011, 15) put it, “carnival time,” and attendees are appreciably in a particularly good mood. Even though, or perhaps because trade fairs are exceptional, they are considerably formalized. Usually, trade fairs take place annually in a designated week of the year. This means that the fairs structure the business year like holidays structure the year of a religious community. All six trade fairs in our sample are a fixture in the business year. Those events are a must that are visited by professionals every year. For those who are interested in the business of children’s books, Bologna Children’s Book Fair is the place to be every year; bookers, event managers, and labels go to Eurosonic in Groningen each January; Europe-based buyers of fictional content for television are likely to start the year in February with EFM/Berlinale, continue in April with MIPTV in Cannes, and travel to Los Angeles to attend the “L.A. Screenings” in May, to finally come back to Cannes in October for the MIPCOM. In traveling to the fairs, participants more or less do the same kinds of things and practices every year: They take the same flight, for example, on Sunday noon before MIPCOM officially starts on Monday. They meet the same people from other corporations already at the airport while waiting at the gates or in the plane. They stay in the same hotel, meet the same people at the same places (e.g., Caffé Roma in Cannes next to the Palais de Festival), and attend the same in-fair events every year. The events and routines at the trade fair, too, do not fundamentally change from year to year: For example, on Sunday evening the “ZDF Sundowner” takes place at the MIPCOM. This event could be described as a mixture of a meeting and a cocktail party organized by ZDF Enterprises, a subsidiary of the German public-service television broadcaster ZDF, at La Croisette, the famous beachside of Cannes just next to the MIPCOM Palais de Festival exhibition side. For many market participants with relation to ZDF—these are predominantly people from the German-speaking countries Germany, Austria, and Switzerland—visiting the ZDF Sundowner has a long tradition: It is the way to start MIPCOM every year. We have observed this strong spatial and temporal standardization also at other trade fairs, especially at Frankfurter Buchmesse with its high number of receptions and award ceremonies, most of which take place each year at the same time and place.

In Collins’ view, the main outcomes of IRs are a feeling of solidarity, strong bonds, and a shared culture. They provide an emotional and interactional basis for what economic sociologists call the social and cultural embeddedness of economic actions. Our business contacts are fully aware of the *social functions of trade fairs*. In the interviews, they unequivocally expressed the importance of business events for cultivating relationships and strengthening mutual trust. Some of them even call their business partners friends. “There are always the usual suspects from Australia and the usual suspects from America and Canada, who simply travel around and attend these events. Ahm, you really make friends there,” says a music publisher who has been in the music business for many years. Indeed, when walking through the trade fair hall, we saw several attendees cordially greeting each other—even at business meetings.

These intense interactions do not only reinforce close network ties but also help to establish and maintain a shared culture, a common understanding of how to do business in the cultural industries and how to evaluate products. Since the symbols of business cultures are enacted and confirmed in dense, ritualized interaction, the business people are affectively bound to them, much more than if they had only learned them from business journals or training. We ourselves as researchers took on the role of apprentices who were introduced to this special business culture through IRs. So let us have a brief look at interactions with one of our respondents, a buyer in the TV industry, whom we interviewed in advance, made friends with during fieldwork, and met at MIPCOM and EFM several times. While in most conversations she impressed us with her expertise regarding content procurement, providing detailed information about market trends, strategic considerations in trading content, and the legal and structural challenges of deal making, there were also some occasions when we discussed the aesthetic qualities of cultural products and their political relevance. In particular screenings of new films (“X-files,” “Greetings from Fukushima”) were events where we had deep and profound discussions about the films, how we enjoyed them, and whether we see a potential for success in domestic markets. Of course, conversations between business representatives and researchers are different from those between business partners. Nevertheless, they give a good impression of how a shared business culture emerges from IRs at business events. The tropes, narratives, and symbols for describing good practices, the whole jargon of doing business in the cultural industries used en passant in conversations is successively and unconsciously adapted by those taking part in interactions. In using them in emotionally grounded situations, practitioners in the field are bound to it and make them a building block of their professional identity (see Gebesmair et al. 2022 for details on different narratives in the cultural industries). Even we as researchers learned to perform convincingly at trade fairs and were committed to central aspects of this business culture.

### Power and Status Rituals at Content Trade Fairs

Let us now move on to a more detailed and ethnographic analysis of IRs. The trade fairs we visited (as probably most trade fairs in all industries) provide a forum for different kinds of encounters. We discovered two essential types of IRs that can be distinguished in their degrees of sociability and formalization, and in how they reproduce the status and power relations of market participants: negotiations and parties/receptions. In fact, real encounters are always a specific mixture of sociability, formalization, emotional tonality, and status display. For analytical purposes, however, it is reasonable to regard these forms separated as a kind of “ideal types.”

We analyze each type in three steps: We start with an account of the type’s sociable and formal character. Then we continue with vignettes that provide a thick description of a concrete IR we observed in the field. Finally, we describe how status and power are negotiated in these encounters.

#### Negotiations

From an economic perspective, economic transactions are at the center of trade fairs. Usually, deals are not closed there but prepared, detailed, and negotiated. Hence, the focus of these encounters is not sociability but doing business. Nevertheless, they have, as we will see, sociable elements and ritual consequences beyond their economic function. Negotiations at trade fairs are *highly formalized* and have a clear spatial and temporal structure. They take place at separated and sometimes hidden areas of the company’s booths, in special halls such as the Buyers’ Club at MIPCOM or Literary Agents & Scouts Centre of the Frankfurter Buchmesse, where cubicles can be rented for meetings, and in special lounges, but also in restaurants, hotel suits, private offices or mansions off-site. Thirty-minute face-to-face meetings are the cross-industry standard practice of interaction between a buyer and a seller of content or services at trade fairs in the cultural industries. These meetings are prearranged far in advance; some of the interview partners reported that they have more than 50 meetings during their three-and-a-half-day stay at the trade fair.

Negotiations often take place in arcane areas and our informants from the industry were very reluctant to let us take part in these meetings. Nevertheless, we could observe negotiations from a greater distance at booths, in only partly covered cubicles, or at lounges. We watched business partners—normally just two—bowing over a staple of sheets and catalogues or looking at a digital display, who appeared to be focused on their tasks and usually show little emotions. Nevertheless, their conversations were accompanied by gestures, certain facial expressions, sudden changes in voice volume, and even laughter, which is typical for these forms of interactions. The businesspeople we interviewed referred several times to the relevance of jokes in these situations. Especially the Viennese are proud of having a special type of humor (the “Wiener Schmäh”), which combines friendliness and wittiness with a whiff of guile what seems especially useful in economic transactions. Others told us how important sympathy is in business interactions and “being on the same wavelength,” as a seller of music services put it. In any case, a large amount of EE is invested in all these meetings, as shown in the following vignette:

On the second day of the Frankfurter Buchmesse, I strolled to the booth of an Austrian publisher to look for Angelika.^4^ I was in luck: she took a short break between two meetings and invited me to join her for a cup of coffee and a cigarette at the venue’s balcony, where we escaped the bustle and noise of the trade fair halls. I have known Angelika since we both studied at Vienna University, but we haven’t met for years. She is now the CEO of one of the largest book publishers in Austria and each year at the Frankfurter Buchmesse as a seller of licenses to international publisher as well as a buyer of rights on content by young non-established authors. Our research project was a good occasion to contact her again after so many years. Our relationship was still cordially and, hence, she didn’t hesitate to complain about the enormous workload at the trade fair. It was early afternoon, and she had just finished her fifth meeting in a row. “It’s hell,” she said and she looked very tired. As so often during fieldwork, I felt a little snoopy and annoying asking her how the meeting went and whether she was successful, since I knew that negotiations were confidential and she longed for a break from business. After 10 minutes, we returned to her booths where the next business partner was already waiting. I was surprised by how quickly she switched back to a professional tone, reactivated all her energy, and greeted the partner very friendly. They took place at a separate table at the booths, where she offered him something to drink. I couldn’t hear them anymore, but I guess there was some small talk before the business partner bowed down to open his briefcase. Since I couldn’t properly hide, I stopped observing them.

We learn from this episode how EE is invested in IRs in order to evoke those feelings between business partners that are a prerequisite for trustful enduring relationships. Although very tired, the moment Angelika approached her business partner she seemed to be in that cheerful mood, which is typical for negotiations. But how is EE confirmed or redistributed and how does it *contribute to stratification*? We have argued in accordance with Collins that all interactions include elements of status and power rituals. This also holds true, especially for negotiations. In negotiations, the status elements and the power elements are subtly entangled with each other. Consider the following description of a business meeting in which one of the research team could take part:

Usually, negotiations are confidential and we were not allowed to participate. Thus, when Robert asked me to accompany him to a negotiation with an international agent I was very glad about this opportunity. Robert is a publisher of high quality, bibliophilic books in small editions, and he is a special kind of character, I had a lot of fun with. This meeting was very important for him, since he hoped to get the German translation rights of an international bestseller. But he felt uneasy for two reasons: First, the meeting was not with his long-time industry contact Monica but with Susan, who replaced Monica and he didn’t know her. Second, the meeting was in English, a language that Robert didn’t speak fluently. Robert was very proud of his charm and his “Wiener Schmäh” but he feared that he would not be able to fully realize this potential in English. Insofar, he was glad to have me by his side to help him out with English.

The meeting took place in the Literary Agents & Scouts Centre at the Frankfurter Buchmesse, called “Forbidden City” by insiders, a huge, guarded hall with dozens of cubicles which can be rented for meetings in 30-minutes slots. Although Robert and Susan met for the first time, greetings were very personal and kisses were exchanged right away. Then they began a little introductory small talk, but everyone seems to be aware that time is very short. Therefore, Bob immediately comes to the issues he wants to bring up. He had prepared himself very well for the meeting and mastered the conversation without any serious problems. Nevertheless, Susan was very formal. Friendly, but formal. She noted everything down on a pad by hand. Finally, she said she will forward this to the rights holder and make inquiries about this. When we left, Robert rushed to Monica, who he discovered at another table during the meeting. This was very important to him, perhaps to show the new business partner at the agency that there already exists a good relationship to the agency and he is a reliable business partner.

So how is status and power renegotiated in this IR? The conversation was clearly focused on business concerns and the mood was very formal during the whole meeting. Nevertheless, there were some emotional investments that had an impact on status and power. On one hand, by kissing each other, gossiping briefly at the beginning, and suggesting closeness through a mutual friend after the meeting, the publisher was eager to express membership in a status group and to establish a trustful relationship. The conversation took place in an atmosphere that enabled interaction at eye level, hence both could acquire EE from this meeting that bound them together. On the other hand, we see that the emotional investments of the publisher were much higher than those of the agent and flew from subordinate buyer to more powerful seller. While the agent stayed calm, concentrated, and to the point during the whole meeting, the publisher tried to impress the agent by being especially casual. Unfortunately, he could not fully apply this emotional strategy in English in the same way he could have done in his mother tongue.

This uneven distribution of EE seems to be a basic feature of business conversations. At several occasions, we observed two business partners in negotiations: one showing little emotion, the other expressively persuading his or her interlocutor to arrive at a reasonable deal. It is not always the buyer who must persuade the seller. Small sellers with little market power also have to invest much energy to get a license for an affordable price. As Beckert (2002) argued, trust in economic transactions must be performatively produced by the trust-taker, that is, the business partner who is in need of being accepted as a trustful business partner. And we would add that in doing so, EE is exchanged between the powerless and the powerful.^5^

#### Parties and receptions

Let us now turn to the other ideal type. Buyers and sellers spend the most time of their stay on negotiations. They rarely attend other events such as panel discussions, lectures, or presentations—except for one: parties and receptions. Parties and receptions are a central element at trade fairs. They usually take place after hours at booths or halls, but especially off-site: in party tents, event locations, museums, hotels, private mansions, or open air. Some of them can be visited with an ordinary trade fair patch, but most of them are by-invitation-only. Through our industry contacts, we had the opportunity to take part in some of these events. There seems to be a standard choreography especially for receptions: after some curbed conversation of entering people, they usually begin with one or more speeches by prominent businesspeople or politicians; then food and drinks are offered, and finally people walk around to meet business partners and friends for small talk—sometimes accompanied by live music.

Through the course of a party or reception, the attention of participants is permanently redirected: from one conversational partner to the other, from conversational partners to the host, to speakers and sometimes also to artists or bands, and back to conversational partners. These kinds of conversations engender, as Collin writes, “feelings of enjoyment and conviviality, and thus (are) satisfying in (themselves)” (Collins and Sanderson 2009, 81). This means that sociability is the main focus of these events and emotions are usually strong and throughout positive, reinforced by considerable consumption of alcohol. Let us have a closer look at one of these receptions:

Michaela, a content buyer for a large TV company, is one of our most important informant and business contacts in our research project. She invited me to meet her at the company’s booth at MIPCOM and I passed by as often as possible to look for her. She was very busy and it was very hard to get into contact with her. I always felt that I was bothering her. But on the second day she invited me to join her for the prestigious “Beta Lunch” at Hotel Barrière Le Majestic next to the Palais des Festivals et des Congrès de Cannes. Of course, by this invitation she was demonstrating her status in the field since the entrance was limited to a selected circle. Hence, I was a little ashamed but simultaneously extremely happy to have the opportunity to attend the reception AND to be accepted as a legitimate apprentice.

The “Beta Lunch” takes place each year on Tuesday at 2 p.m. and is hosted by Jan Mojto, one of the largest film producers and rights owners in Europe. I met Michaela and her assistant in front of the hotel and together we entered a very glamorous hall of the hotel through a side door. Shortly after we had entered the hall, Michaela introduced me to a middle aged, casually-dressed man, who turns out to be a very important content seller of a competing company. Michaela briefly talked to him, while the assistant and me stayed aloof, enjoying the festive mood in the hall. After the crowd of an estimated two or three hundred invited guests had filled the hall, a perfectly produced video with trailers of Beta’s newest productions was shown and the people became silent, devoutly watching the presentation. Immediately after the screening, the host Jan Mojto continued with a witty speech peppered with irony and allusions that provided much material for the conversations afterwards among the guests. During this event, I was introduced to several other businesspeople who shared their opinions on the Beta productions as well as the on the event with me. They all stressed how important these events and the glamorous environment is. “People are in much better mood for negotiations in such places”, another representative of a TV company said. I was very happy to be addressed as an equal conversation partner, although they knew that I was a researcher and not a businessperson. By and by, the conversations became more business-related and some attendees began to walk around in search for relevant business partners. Michaela headed for the content seller we met at entrance immediately after Mojto’s speech. She was obviously interested in one of their productions and stretched out her feelers. I was astonished how enjoyment and business intermingle at these occasions. After an hour, Michaela and her assistant clearly indicated to me that they had to leave for business meetings and I could not join them anymore.

How are status and power plays intermingled in this ritual? The group of guests at this prestigious event doubtlessly form a status group, which is clearly marked off against outsiders not just by access barriers (by-invitation-only) but also by cultural resources which comprise a specific knowledge (about the newest releases and available content, Motjo’s role in the industry, and the persons he alluded to in his speech) and specific forms of demeanor (pecks on both cheeks when greeting, laughing out loud in the right moment). The more the attendees talk to each other, the more they are emotionally entrenched and develop a feeling of group solidarity. Additionally, the more they are recognized and addressed by others, and the more they can impress others with stimulating stories, the more they develop a feeling of self-esteem and confidence. In Collins’ terms: EE is redistributed. Those in the center of ceremonies, in this case, the host Jan Mojto, have the highest chance to accumulate large amounts of this energy, which is reinvested in future encounters.^6^

We ourselves as researchers took part in these status and power rituals and could feel how EE is distributed at these occasions. Since our industry contacts introduced us to their business partners, we experienced what it was like to be included in a status group. We got an impression of the collective effervescence when one takes part in an exclusive reception, in glamorous hotel halls with food and drinks, and stimulating conversation. But we also learned how it feels to be at the margins of the field and how much effort is needed to approach people you do not know to start a conversation. And in the moment when we were left behind by your business contacts, we felt all the sadness of not really being one of them and of being relegated again to our position as just unimportant researchers.

----

There are many other types of events at trade fairs such as award ceremonies, screenings, concerts, and readings, but also panel discussions, business lectures, and demonstrations of new technologies (see Figure 1). We observed special networking events for newcomers and less established firms—first-timers’ breakfasts, matchmaking lounges, speed meetings, startup competitions, meet the speakers, artist accelerators—and also space for spontaneous encounters: at booths, in corridors, at food shops, at coffee lounges, etc. They differ in their degrees of formalization and sociability and they play different roles in the process of stratification. While, for example, spontaneous encounters can be considerably sociable but are little formalized (except for the subtle forms of demeanor Goffman has analyzed), award ceremonies are usually scripted but leave only a little space for sociability (in contrast to the parties that usually follow the ceremony).

The uneven distribution of EE and the entanglement of status and power rituals are especially visible in interactions between newcomers and incumbents. As mentioned above, there are many formal and informal ways for newcomers to approach established representatives at trade fairs. Especially the most powerless often cue up to get an appointment with a panel speaker or a company representative. At the Children’s Book Fair in Bologna as well as at the Frankfurter Buchmesse, we saw long lines of illustrators waiting for a short meeting with a publisher to present a collection of their illustrations. This can certainly be a humiliating experience and most of them looked somehow distressed and strained. Nevertheless, these or other meetings can be an entry point to the market. In fact, we met young aspirants who succeeded in making the acquaintance with a relevant businessperson at meetings, parties, or just at the company stand. A friendly conversation, in which experienced office holders give advice to newcomers, could substantially increase the newcomer’s self-esteem—and be the first step toward a career in the cultural industries. Again, maintaining power relations (between established executives and newcomers) and providing access to a status group are tightly interwoven in IRs.^7^

## Discussion

So far, we followed Collins’ theory of IR, which proved to be a valuable instrument for analyzing interactions and conversations at trade fairs. However, there are two aspects in which our view deviates from Collins’ approach: the role of economic resources and the role of strategies in IRs. We begin with the first.

In Collins’ perspective, there are only two relevant resources for IRs: *cultural resources* and *emotional energy*, which are reproduced and redistributed throughout chains of rituals (Collins 1979, 1981, 2004; Collins and Sanderson 2009). Without doubt, cultural resources—also called conversational resources or membership symbols by Collins—play an important role in IRs, as we have observed during fieldwork: there is a specific (rather casual) dress code, a certain manner of greeting and speaking, there is a particularized knowledge on business gossips and a more generalized knowledge on recent releases and upcoming artists, and, of course, all of the actors know how to bargain and make a deal (with all its contractual subtleties). Emotions are a crucial element of IRs as well. We saw excitement, enthusiasm, joy, confidence, content, but also strain, uneasiness, sadness, and boredom. Collins is a little vague in his definition of EE, which is based on an ethological interpretation of human interaction (Collins and Sanderson 2009, 55–64): Like animals, humans affect each other emotionally when interacting and leave conversations in a positive or negative mood. Nevertheless, we agree with Collins and others (Bandelj 2009, 2015; Berezin 2009; Pixley et al. 2014) that emotions play an important role in establishing a shared culture and in (re-)producing stratifications—in material markets as well as in other parts of the society.

But how do *economic resources* affect IRs and the outcome of IRs? Collins (1993, 2004, chapter 4) highlights two aspects: First, they provide the material basis for IRs. In order to take part in interactions at trade fairs, the business partners must invest enormous amounts of money. Entrance fees, costs for the company booths, expenses for food, drinks, and entertainment at parties, plus travel and accommodation in Cannes’ most expensive hotels add up to six-digit euro amounts for a large company. In the past, the largest media corporations tried to outperform each other with luxurious trade fair appearances in a potlatch-like race.^8^ Even the smallest companies must invest a considerable part of their marketing budget in trade fairs. Second, economic resources become the focus of rituals themselves. Collins assumes that in some parts of contemporary society, money became a “sacred object,” which is ritually worshipped and loaded up with strong emotions. Although this may hold true, especially for financial markets, we did not observe this kind of excessive invocation of financial resources in the cultural industries. In contrast, actors in the field of cultural production try to appear not too profit-oriented (Bourdieu 1983).

However, we think that Collins underestimates the direct impact of the uneven distribution of economic resources on IRs. Business partners in trade fair interactions represent firms with a certain market power, defined by the degree of competition in a market and the dependency on relevant resources (Pfeffer and Salancik 2003; Porter 1998).^9^ This market power is not only translated into EE through repeatedly successful interactions but also directly structures conversations. A buyer or seller of licenses *knows* how powerful a business partner is and behaves accordingly. As Turner (2019) has argued in his critique of Collins’ approach, the macro and meso levels of society have an immediate effect on interactions at the micro-level of personal encounters by defining the expectations of interaction partners (see also Wherry 2014, 429). Business partners are usually well informed about the relative market power of their partners. As we have seen, there are corresponding expectations about who has to pay deference to whom, how one has to behave in negotiations, and who has to invest more EE to persuade the other to give his or her consent to an arrangement. Insofar, we regard not only cultural resources and EE as crucial elements of IRs but also the uneven distribution of financial resources and market power.

This brings us to another aspect in Collins’ theory. In his view, interaction partners emotionally entrain each other during conversation. The more EE and cultural resources they bring to an encounter, the more they can dominate an interaction. Through this (subliminal) process of mutual affection, they implicitly maximize their EE (Collins 1993, 2004, chapter 4). However, as Rössel (1999, 31) pointed out in his discussion of Collins’ conflict sociology, people must satisfy their basic needs beyond EE—a notion that is left out in the theory of IRs. Humans also aim at maximizing their individual benefits from interactions or at least satisficing their needs and, hence, *instrumentally use emotions in order to achieve certain goals*. Participants in cultural markets (as in all other commercial markets) obviously strive for maximizing profits from transactions. Therefore, IRs are always a stage for contest where emotions are strategically deployed in order to achieve certain goals. In our fieldwork we discovered a lot of strategic use of IRs: The way in which newcomers, as well as established executives, strategically position themselves at parties to “run into” an important business partner, the forms of arousing trust, impressing conversational partners, and persuading them to agree to a deal, the placement of jokes that relaxes a strained situation, the expression of joy and excitement about products that confirms an affinity in taste and orientation, in all these situations emotions, play an instrumental role to achieve goals beyond emotional wellbeing. Of course, we cannot fully control our emotions, often we are simply overwhelmed by them (Bandelj 2009, 359). Nevertheless, we believe that to fully understand the role of IRs in economic action (as well as in other areas), we should conceive of rituals not just as unconscious emotional entrainment but also as a field of strategic mastering of emotions. Therefore, in IRs we obviously oscillate between emotional entrainment and strategic choice rather than simply following a ritual script.^10^

## Conclusions

In this article, we analyzed different interactions between business partners at content trade fairs. These interactions differ in their degrees of formalization and sociability and they play an important role in the stratification of the field. Emotions play an important role in all these encounters and help to bind business partners to each other as well as to develop a shared understanding of their business. Following Randall Collins’ microtheory, we believe that IRs are crucial to the social and cultural embedding of economic actions. They contribute not only to stable ties, a feeling of solidarity, and shared symbols in a market, they also (re-)produce boundaries and inequalities. The more EE market participants are able to accumulate through chains of IRs, the higher they rise in the system of social stratification.

Although we do not question the relevance of emotions to bolster status and reinforce power relations, we suggest expanding Collins’ view of resources and strategic action. The business partners in our fieldwork had a clear understanding of their own and their partner’s market power and behaved accordingly. The less powerful had to invest much more EE than those in a more advantageous market position. Meaning that financial resources and power relations structure the interaction independently of the relative amount of EE. They are anticipated by interaction partners. Furthermore, there seems to be a permanent switch between emotional entrainment and strategic action. Of course, in some situations, emotions have a firm grip on us but simultaneously we produce emotions in order to achieve certain goals. At trade fairs, we often observed the instrumental use of jokes and small talk to evoke a comfortable mood in negotiations or other types of business conversations. This strategic production of emotions seems to be a crucial element in economic transactions and must not be neglected in the analysis of IRs, especially in a market context.

Based on our insights from fieldwork in the cultural industries, we see several lines of further research. First, the balance of emotions and cognitions, the alternation of entrainment, and strategic manipulation of emotions deserves more research. Perhaps, we can conceive IRs as scripts (Abelson 1981) which include a kind of “escape button” or sequences of deliberate as well as automatic cognition (DiMaggio 1997). Therefore, a much more detailed conversation analysis of business talks than we were able to provide is required. How is the process of turn-taking, a central aspect in ethnomethodology as well as in Collins’ theory, organized in negotiations between business partners? Second, we analyzed only single IRs, but not a whole chain of interactions. Hence, it might be interesting how positions are changed or just reinforced in sequences of interactions. Accompanying a market participant for a longer period could provide further insights into how inequalities in markets are transformed, bearing in mind that there are certainly many methodological problems in this endeavor (field access, confidentiality, etc.). Finally, further research must clarify if our results can be applied to other industries. The cultural industries are doubtlessly much more “personal” and “emotional” than others. In contrast to other industries, the goods and services themselves bring a lot of glamour, and thus, high spirits to the trade fairs. A trade fair where actors, directors, musicians, and writers are important ingredients is definitely more celebratory than a trade fair for capital goods. Nevertheless, we know from other industries that they also invest enormous amounts of money in booths, ceremonies, and parties during trade fairs and, therefore, we believe that those IRs we have analyzed in our project can be found also in other industries.

## Notes

1. We discuss the cultural function of business events at length in Gebesmair et al. (2022).

2. We are writing this article amid the COVID-19 pandemic, which radically reduced bodily copresence in all parts of society all over the world. There have been some discussions, also in pre-pandemic times, as to whether bodily copresence is a prerequisite of IR (see for example Knorr Cetina and Bruegger 2002; DiMaggio et al. 2019). Geographers have argued that computer-mediated communication even outperforms face-to-face communication in some business contexts (Bathelt and Turi 2011) and many trade fair operators successfully organized their business events online in 2020 (see Ebner-Zarl 2020 on the Frankfurter Buchmesse). Nevertheless, we believe that virtual meetings will never have the same ritual strength as face-to-face encounters and therefore might erode social cohesion in the long run (see Collins 2004, 53–64).

3. We thank a reviewer for reminding us of Simmel’s seminal contribution to this subject.

4. All names are changed for anonymization.

5. Furnari and Rolbin (2018, 31) similarly described how managers of creative projects act emotionally to excite funders to commit their resources.

6. Trade fairs provide a perfect opportunity to display market power (Entwistle and Rocamora 2011; Havens 2011; Moeran and Pedersen 2011). The large media companies invest enormous amounts of money to impress market participants with fancy booths sometimes larger than basketball fields. In contrast to the authors above, we are not interested in how power is demonstrated at booths through material value and conspicuous consumption, but especially in how market power translates into EE in IRs.

7. There are several other power rituals between businesspeople and subordinates such as, for example, service personal or assistants. Since our focus is on economic transactions on cultural markets, we do not elaborate on these.

8. Informants reported about prominent firms that withdraw from trade fairs for economic reasons—with fatal consequences for their reputation.

9. Market power is neither defined by the size of a company nor by its position in a value chain, but solely by competition and scarceness of resources. Even a relatively small company such as the Austrian Broadcasting Corporation exercised some market power on global content markets, since it formed a monopsony in the 1980s (see Wildman and Sivek 1988 for an economic explanation of power in global cultural markets). After the liberalization of the Austrian TV market at the turn of the century, it lost power.

10. Arlie Hochschild has shown in her seminal work on emotions that we are not simply overwhelmed by feelings but consciously manage them in order to comply with so called feeling rules (see e.g., Hochschild 1979). In contrast to Hochschild who focuses on the strategic manipulation of one’s own emotions, we are more interested in the deliberate management of mood in a situation and its effects on others.
